# Symptoms, burden, and unmet needs of patients living with exocrine pancreatic insufficiency: a narrative review of the patient experience

**DOI:** 10.1186/s12876-024-03188-w

**Published:** 2024-03-14

**Authors:** Jodie A. Barkin, Trudi B. Delk, Valerie J. Powell

**Affiliations:** 1 University of Miami Miller School of Medicine, 1120 NW 14th St., Clinical Research Building, Suite 1188 (D-49), 33136 Miami, FL USA; 2 Aimmune Therapeutics, a Nestlé Health Science Company, Brisbane, CA USA; 3 CorEvitas, LLC, part of Thermo Fisher Scientific, Waltham, MA USA

**Keywords:** Exocrine pancreatic insufficiency, Pancreatic enzyme replacement therapy, Pancreatic enzymes, Patient experience

## Abstract

Exocrine pancreatic insufficiency (EPI) stems from a deficiency of functional pancreatic enzymes with consequent maldigestion and malnutrition. EPI shares clinical symptoms and manifestations with other disorders and is a considerable burden to individuals affected. In this narrative review, we analyzed the literature to identify relevant publications on living with EPI with the scope of individuating evidence gaps, including those related to symptoms, health-related quality of life (HRQoL), emotional functioning, disease burden, presence of comorbidities, and the use of pancreatic enzyme replacement therapy (PERT). Abdominal pain emerged as one of the most prominent symptoms. HRQoL was affected in EPI, but no articles examined emotional functioning. Comorbidities reported involved other pancreatic disorders, diabetes, gastrointestinal disorders, sarcopenia and osteopenia, cardiovascular disorders, bacterial overgrowth, and nutritional deficiencies. PERT was found to be effective in improving EPI symptoms and was well tolerated by most individuals. Our review revealed a dearth of literature evidence on patients’ experience with EPI, such as emotional functioning and disease burden. We also revealed that studies on long-term effects of PERT are missing, as are studies that would help advance the understanding of the disease and its progression, risk/mitigating factors, and comorbidities. Future studies should address these identified gaps.

## Background

Exocrine pancreatic insufficiency (EPI) is characterized by maldigestion of macronutrients and micronutrients resulting from a relative lack of functional pancreatic enzymes due to reduction in synthesis or secretion, lack of mixing, or inactivation of pancreatic digestive enzymes. Key symptoms of EPI can include steatorrhea, weight loss, vitamin deficiencies, and malnutrition [1]. Although overt maldigestion is associated with obvious symptoms and impact on quality of life [2], long-term malnutrition can have significant detrimental consequences on overall health, as well as increased risk of mortality in patients with chronic pancreatitis (CP) due to an increased overall risk of cancers, infections, and cardiovascular events [3, 4]. Thus, it is important that EPI be diagnosed early and treated appropriately to alleviate symptoms and reduce the risk of long-term complications. However, EPI symptoms and manifestations are not specific and are often shared with other gastrointestinal (GI) conditions; this can lead to a lack of recognition or improper diagnosis in many individuals with EPI. Of those who are correctly diagnosed, many are not treated with appropriate dosages of pancreatic enzyme replacement therapy (PERT) [5–9]. Accordingly, many patients continue to experience a myriad of symptoms, which result in a high level of disease burden in this population.

We performed a search of the scholarly literature to identify published articles on the patient experience of adults living with EPI, specifically pertaining to symptoms such as abdominal pain and GI symptoms, health-related quality of life (HRQoL), emotional functioning (e.g., anxiety and depression), overall disease burden, and unmet needs. We also sought to identify articles on comorbidities associated with EPI. A final focus area of this search was the use of PERT for the treatment of EPI, as treatment of this condition may have an impact on symptoms and overall patient experience. Articles included in this review mainly focused on adult patients with EPI; however, if a study had both adult and pediatric patients, it may have been included if the majority of patients were adults.

## Patient experience

### Abdominal pain

A literature search revealed that abdominal pain is one of the most prominent symptoms reported by individuals suffering from EPI; yet, to date, the amount of published literature on abdominal pain in individuals with EPI is limited [2, 10–19]. It may be challenging to differentiate abdominal pain due to EPI versus abdominal pain due to underlying CP because of the multifactorial nature of pain in this patient population.

One study that conducted qualitative interviews with individuals with EPI confirmed that abdominal pain is one of the key symptoms associated with EPI, with 84% of included participants reporting having experienced abdominal pain, 96% of them spontaneously [2]. Of those, 78% described abdominal pain occurring mostly in their stomach. Patients with cystic fibrosis (CF) most often reported pain in the upper and lower abdominal quadrants, whereas patients with CP more often reported upper right abdominal pain or epigastric abdominal pain. Both patients with CF and CP also reported nonabdominal pain, but the authors concluded that it was unclear whether that pain should be attributed to EPI.

Two open-label studies showed that PERT leads to improvements in EPI-related symptoms, such as abdominal pain, stool consistency, and flatulence [12, 14]. Furthermore, another randomized controlled study showed improvements in abdominal pain in patients who were on PERT compared to patients who were on placebo [18], whereas a randomized controlled trial comparing one PERT formulation (pancrelipase, Creon®) to placebo showed that patients in both treatment groups improved in their abdominal pain [17]. Finally, a randomized, placebo-controlled crossover trial showed that PERT improved abdominal pain compared to placebo [20]. In turn, improvements in abdominal pain and GI symptoms have been shown to improve quality of life [10, 21].

### GI symptoms

In the literature, the most commonly reported GI symptoms included bloating, flatulence, bowel movement/stool symptoms, dietary symptoms, and to a lesser degree, nausea and vomiting [2]. Among bowel movement/stool symptoms, the most common symptoms appeared to be diarrhea, steatorrhea, foul-smelling stools, and increased stool frequency and urgency [2].

Multiple studies on PERT have demonstrated that PERT significantly improves fat absorption, along with a significant reduction in fat excretion, and improves stool frequency and consistency [12, 14, 17, 18, 22, 23]. However, in severe pancreatic insufficiency, steatorrhea may be challenging to fully resolve even with appropriately dosed PERT due to the complex nature of the digestive process and limitations in exogenous supplementation, by which PERT may only achieve a 60 to 70% reduction in fat maldigestion in certain situations [24].

In addition to PERT use, patients are advised to modify their dietary and lifestyle habits to manage their symptoms, including cessation of smoking and alcohol consumption to not further damage their pancreas, and dietary consultation [25]. Historically, a low-fat diet has been recommended in EPI to reduce steatorrhea; however, this recommendation has been abandoned in modern dietary counseling in EPI due to the risk of aggravating EPI-related weight loss and deficiencies of lipid-soluble vitamins [26]. Dietary consultation should include advice for sufficient caloric intake and normal fat content.

### HRQoL

HRQoL is a multidimensional concept that includes domains related to physical, mental, emotional, and social health and is commonly used to examine the impact of health status on quality of life. An increasing number of people live with chronic diseases that can adversely affect their HRQoL. HRQoL measures are important to evaluate the impact of a disease and the effects of medical intervention, and improvements are often used to gauge effectiveness of administered therapies [27].

Improvements in HRQoL are considered to be essential determinants of therapeutic benefit [27]. Patients with CP experience substantial impairments in HRQoL. A retrospective analysis showed that severity of abdominal pain, chronic pancreatic diarrhea/steatorrhea, low body weight, and loss of work independently contributed to the physical component score of the 36-Item Short Form Health Survey (SF-36). These also were the factors most closely associated with poor health status perception, whereas the etiology and duration of the disease or changes in pancreatic morphology had no impact on HRQoL [28]. Similarly, studies on EPI have shown that HRQoL is adversely affected by EPI and its significant symptoms and EPI impacts all HRQoL domains [2, 29].

Evidence from studies indicates that PERT improves HRQoL [10, 14, 21]. However, other studies have failed to document an improvement of HRQoL in patients who experience symptom improvements as a result of PERT [12, 30]. The reason for these discrepant findings remains unknown; however, it is likely due to the observation that many patients still experience symptoms despite PERT use, and perhaps due to the complex and multifactorial nature of pain in CP where there may be overlap of pain triggers not wholly attributed to EPI alone. Alternatively, the selection of the HRQoL instrument may have been a contributing factor because generic HRQoL questionnaires (such as the SF-36) may not be sensitive enough to detect more subtle, disease-specific changes in this population.

### Comorbidities

Literature indicates that individuals with EPI exhibit a wide range of comorbid disorders. The most common comorbidities include other pancreatic diseases such as severe/acute pancreatitis [31–34], chronic pancreatitis [3, 35, 36], pancreatic resections and other pancreatic interventions [37–46], diabetes [47–59], and other GI disorders such as irritable bowel syndrome and celiac disease [60–62], although potentially all of these may be the underlying cause for EPI.

Studies also have found a relationship between EPI and sarcopenia and osteopenia [63–65], likely occurring as a consequence of prolonged malnutrition and malabsorption due to EPI. Conversely, a study has shown that patients who are on PERT had significantly higher bone mineral density as measured by dual-energy X-ray absorption [66].

Furthermore, EPI has been linked to cardiovascular disorders [4], small intestinal bacterial overgrowth [67, 68], and nutritional deficiencies [69, 70], which also may contribute to higher mortality and morbidity rates in patients with untreated EPI [3, 26].

Finally, case reports were identified that describe the occurrence of EPI in a variety of other disease states [71–77].

### PERT

Both interventional and observational studies were identified in the literature on the effects of PERT in EPI. A total of 20 studies of prospective, double-blind, placebo-controlled trials [10, 11, 14, 15, 17–20, 22, 23, 78–87] and an additional 5 open-label studies [12, 88–91] were noted and examined.

The most commonly used efficacy endpoints in the examined studies were changes in coefficient of fat absorption, body weight, HRQoL measures, and clinical symptoms (e.g., steatorrhea, stool frequency and consistency, abdominal pain, flatulence, etc.), all of which were included as metrics of absorption improvements due to enzyme replacement. Safety assessments involved the recording of treatment-emergent adverse events (TEAEs) in most studies. Studies did not report any serious adverse events, and the most common TEAEs were identified as stomach pain, nausea, bloating, headache, and dizziness. Although hyperuricemia was reported in patients with CF treated with pancreatic enzyme products [92], more recent studies did not find a significant association between the use of pancrelipase and hyperuricemia [22, 84].

The literature shows that PERT is overall effective in improving EPI symptoms and is well tolerated by most individuals. Nevertheless, the main limitation of the literature identified is that most studies enrolled relatively small numbers of participants, and for most studies, the treatment duration was short (often less than a few weeks in length). Evidence from two longer-term studies showed long-term safety and efficacy for PERT in individuals with EPI due to CP or pancreatic surgery [12, 21]; however, more evidence on the long-term effects of PERT on EPI, and particularly real-world evidence, is needed to document the impact of PERT on EPI.

## Discussion

Literature on several aspects of EPI, specifically the patient experience in EPI (i.e., symptoms, HRQoL, overall disease burden, and unmet needs), comorbidities that have been associated with EPI, and PERT for the treatment of EPI has been published (Table 1). Recent publications have also shown that patients actively search for information on EPI and that a proportion of patients have modified or stopped PERT of their own accord for a variety of reasons [93–95]. To date, information is lacking evidence on the patient experience around EPI, particularly emotional functioning and disease burden. Additionally, individuals with EPI have a myriad of comorbid disorders, which may confound the clinical presentation of EPI as well as make assessing response to therapy with PERT a challenge. For individuals with EPI on treatment with PERT, there remains scarce evidence on the effects of PERT therapy on conditions comorbid to EPI such as emotional functioning and HRQoL.

**Table 1:** Summary of key findings in the literature

Topic of interest	Key findings
Abdominal pain	• Abdominal pain is a frequently reported symptom (e.g., reported by 84% of patients in 1 study)
GI symptoms	• Most commonly reported GI symptoms are bloating (64%), flatulence (33%), bowel movement/stool symptoms (e.g., diarrhea [75%], change in stool color [51%], fatty stools [49%], constipation [48%], bowel urgency [33%]), and dietary symptoms (e.g., weight loss [67%], loss of appetite [33%]) • Steatorrhea can only generally be reduced by 60–70% using PERT
HRQoL	• Severity of abdominal pain, chronic pancreatic diarrhea, low body weight, and loss of work independently contributed to the physical component score of the Short Form-36 (adjusted *R*^2^ = 33.8%)
Emotional functioning	• No articles related to emotional functioning related to EPI were located
Comorbidities	• Most common comorbidities included diabetes, pancreatic resections and other pancreatic interventions, severe/acute pancreatitis, chronic pancreatitis, and other GI disorders
PERT	• Commonly used efficacy endpoints included coefficient of fat absorption, body weight, HRQoL measures, and clinical symptoms

*Abbreviations* EPI, exocrine pancreatic insufficiency; GI, gastrointestinal; HRQoL, health-related quality of life; PERT, pancreatic enzyme replacement therapy

Fibrosing colonopathy is the only serious complication of PERT use primarily at much higher doses than are used in the vast majority of patients and can develop several months to several years after starting high-dose PERT [96]. Although the pathogenesis of fibrosing colonopathy is unknown, it is highly correlated with abdominal pain, flatulence, and headache, and is experienced by few study participants. Because conflicting/controversial data have been published on this topic, further discussion of this rare but catastrophic potential complication is beyond the scope of this review.

There is complex overlap of abdominal pain etiologies in patients with both CP and EPI, given the multifactorial nature of pain in these conditions. Although EPI is treated with PERT, for individuals with pain in CP, therapies may include endoscopic interventions, opioid and non-opioid analgesics, and/or surgery. Standard treatment for EPI consists of PERT along with dietary interventions. Several pharmacotherapy studies have been published with individuals with CP [12, 18, 22, 23]. These studies revealed that PERT improved clinical symptoms; however, given that the mortality rate of patients with CP is three- to four-fold higher than that of the general population, the 20-year survival rate is less than 50% [97]. Clinical studies examining the effects of pancrelipase on long-term mortality are currently lacking, and long-term studies are needed to determine whether PERT reduces the risk of mortality in addition to improving clinical symptoms.

EPI occurs in approximately 85% of patients with CF [98]. CF is a rare autosomal recessive disease that affects pancreatic and lung function from birth [99, 100]. Patients with CF have a buildup of thick mucus in their lungs and pancreas which results in dyspnea and blockages in the secretion of pancreatic enzymes. The standard CF treatment regimen includes chest physical therapy, mucolytics, aerosolized antibiotics, and PERT [99, 100]. In addition, patients with CF are advised to increase their consumption of calories, protein, fat, and appropriate minerals and vitamins. Patients with CF are at increased risk of malnutrition as a result of nutrient malabsorption; children who have poor nutritional status may experience impaired growth [100]. Thus, PERT is necessary in children and young patients with CF to ensure proper growth. Double-blind, randomized, placebo-controlled studies and an open-label study have shown that PERT treatment improves fat and nitrogen absorption and clinical symptoms in patients with CF compared with placebo, with favorable safety profiles [11, 20, 84, 101].

Patients who have a partial or total pancreatectomy experience EPI due to a reduction or total loss of pancreatic tissue; PERT is essential in these patients to replenish the loss of pancreatic enzymes and maintain adequate digestion. Patients with CP who have intolerable pain, evidence of necrosis in pancreatic tissue, or a pancreatic tumor may undergo a partial pancreatectomy. Patients who have EPI due to CP, CF, or pancreatectomy can be treated with pancrelipase products with good tolerability [12, 18].

Another issue regarding the use of PERT is that EPI is a condition that arises from a highly heterogeneous group of diseases. EPI is a result of pancreatic diseases such as CP, pancreatic cancer, and pancreatic surgeries/interventions, as well as extra-pancreatic diseases and conditions such as GI diseases (e.g., inflammatory bowel disease, celiac disease) and diabetes. The prevalence of some of these extra-pancreatic diseases and conditions is rapidly increasing worldwide [102, 103]. Yet, to date, the clinical relevance and application of PERT for these conditions have not been documented. Therefore, additional studies are needed to explore fully the underlying mechanisms and to determine the need for improving nutritional status and effects on morbidity/mortality in people with diabetes or GI diseases, and in the elderly. Also, observational studies with individuals with EPI and comorbid disorders of pancreatic and extra-pancreatic origin would be useful to document the efficacy and safety of PERT in these groups of patients.

Finally, although a number of studies have been identified that have evaluated the efficacy and safety of PERT, long-term studies documenting the effects of PERT are missing, as are studies on measures that could advance the understanding of the disease, comorbidities, disease progression, risk, and/or mitigating factors. Accordingly, future studies are needed to address the above-mentioned gaps by collecting a comprehensive set of data around disease and treatment outcomes.

Only one targeted literature review in the field of EPI is known to be available in the literature [38]. The authors of this review concentrated on EPI resulting from GI surgery, which is only one of the possible causes of EPI. This more focused review led to a conclusion that EPI is difficult to diagnose even in this specific group of patients and underlined the importance of appropriate PERT dosing and patient monitoring [38].

A main limitation of this review is the number of included studies. This narrative review seeks to capture the essence of the patient perspective in EPI through the lens of symptom burden, HRQoL, emotional functioning, overall disease burden, and unmet needs and is not an exhaustive or comprehensive synthesis of the available literature.

## Conclusions

In conclusion, our narrative review confirmed that abdominal pain is the most prominent symptom reported in EPI but may be due to the underlying disease of CP more than EPI itself. The most commonly reported GI symptoms of EPI itself included bloating, flatulence, bowel movement/stool symptoms (i.e., diarrhea, steatorrhea, foul-smelling stools, and increased stool frequency and urgency), dietary symptoms, and to a lesser degree, nausea and vomiting. Unsurprisingly, HRQoL is adversely affected by EPI and its symptoms and EPI impacts all HRQoL domains. Individuals with EPI suffer from many comorbidities, and treatment with PERT led to lessening of abdominal pain, significantly helped fat digestion and absorption, and reduced fat excretion leading to the improvement of stool-related symptoms. Alternatively, there is a paucity of literature concerning emotional functioning and disease burden in EPI. Also, studies on long-term effects of PERT and studies to explain the disease and its progression over time were missing. Such a broad review has not been done to date in EPI and has identified key individuated knowledge gaps in the patient experience in EPI to be addressed in future studies.

## Data Availability

Data source: MEDLINE (via PubMed), Cochrane Database of Systematic Reviews (CDSR), and Embase databases. The datasets used and/or analyzed during the current study are available from the corresponding author upon reasonable request.

## Abbreviations

CF: cystic fibrosis
CP: chronic pancreatitis
EPI: exocrine pancreatic insufficiency
GI: gastrointestinal
HRQoL: health-related quality of life
PERT: pancreatic enzyme replacement therapy
SF-36: 36-Item Short Form Health Survey
TEAE: treatment-emergent adverse event
